# Reprogrammed keratinocytes from elderly type 2 diabetes patients suppress senescence genes to acquire induced pluripotency

**DOI:** 10.18632/aging.100428

**Published:** 2012-02-04

**Authors:** Seiga Ohmine, Karen A. Squillace, Katherine A. Hartjes, Michael C. Deeds, Adam S. Armstrong, Tayaramma Thatava, Toshie Sakuma, Andre Terzic, Yogish Kudva, Yasuhiro Ikeda

**Affiliations:** ^1^ Department of Molecular Medicine, Mayo Clinic, Rochester, MN 55905, USA; ^2^ Cell Therapy, Department of Laboratory Medicine/Pathology, Mayo Clinic, Rochester, MN 55905, USA; ^3^ Robert and Arlene Kogod Center on Aging, Marriott Heart Disease Research Program, Division of Cardiovascular Diseases, Departments of Medicine, Molecular Pharmacology and Experimental Therapeutics, and Medical Genetics, Mayo Clinic, Rochester, MN 55905, USA; ^4^ Division of Endocrinology, Mayo Clinic, Rochester, MN 55905, USA

**Keywords:** rejuvenation, electron microscopy, disease modeling, INK4/ARF locus, caspase, GPX1, PDX1

## Abstract

Nuclear reprogramming enables patient-specific derivation of induced pluripotent stem (iPS) cells from adult tissue. Yet, iPS generation from patients with *type 2 diabetes* (T2D) has not been demonstrated. Here, we report reproducible iPS derivation of epidermal keratinocytes (HK) from elderly T2D patients. Transduced with human OCT4, SOX2, KLF4 and c-MYC stemness factors under serum-free and feeder-free conditions, reprogrammed cells underwent dedifferentiation with mitochondrial restructuring, induction of endogenous pluripotency genes - including NANOG, LIN28, and TERT, and down-regulation of cytoskeletal, MHC class I- and apoptosis-related genes. Notably, derived iPS clones acquired a rejuvenated state, characterized by elongated telomeres and suppressed senescence-related p15^INK4b^/p16^INK4a^ gene expression and oxidative stress signaling. Stepwise guidance with lineage-specifying factors, including Indolactam V and GLP-1, redifferentiated HK-derived iPS clones into insulin-producing islet-like progeny. Thus, in elderly T2D patients, reprogramming of keratinocytes ensures a senescence-privileged status yielding iPS cells proficient for regenerative applications.

## INTRODUCTION

Over 200 million people worldwide, ranging from 20 to 79 years in age, suffer from diabetes mellitus, typically the late onset type 2 diabetes (T2D) [1, 2]. By 2025, this number is projected to rise to over 300 million propelled by the aging of the population. Thus, new treatments for T2D, including approaches to address progressive pancreatic beta cell insufficiency, are needed.

Competent for multilineage differentiation, embryonic stem (ES) cells are regarded potentially promising for regenerative applications. Differentiation of ES cells into transplantable tissues could lead to repair therapies for severe degenerative diseases, including diabetes. In preclinical studies, human ES cells have been differentiated into insulin-producing cells [3-5], which can reverse the course of diabetes [3]. However, ES cell use is associated with ethical issues and further limited by allogeneic mismatch, restricting clinical application. Recently established nuclear reprogramming methodologies allow generation of autologous pluripotent stem cells from somatic sources. Ectopic expression of primordial transcription factors [6-10] dedifferentiates adult somatic tissue into induced pluripotent stem (iPS) cells. In general, iPS cells share similar properties with ES cells with respect to morphology, growth, expression of pluripotency-associated factors, self-renewal and multilineage potential [11]. Analysis of global gene expression profiles of human iPS cells has also revealed patterns similar to those of human ES cells, with notable upregulation of pluripotency-associated genes [12, 13]. Similar to ES cells, human iPS cells can be induced to differentiate into various cell types, including insulin-producing cells [14-17], as well as tissues associated with T2D complications such as neurons [18] and heart muscle [19-21]. Diverse somatic sources have been successfully reprogramed, including fibroblasts, stomach and liver cell cultures [22], and blood cells including mature B and T lymphocytes [23]. Moreover, human keratinocytes [24, 25] are a promising resource for clinical-grade iPS derivation. Indeed, efficient and rapid iPS derivation from keratinocytes has been demonstrated from a 4 year-old child and 28-35 year-old young adults [25]. Disease-specific iPS derivation has been also reported from young adults with various genetic or degenerative diseases, including generation of iPS cells from type 1 diabetes (T1D) [14, 26]. However, to date, derivation of iPS cells from T2D patients has not been documented. Moreover, it remains uncertain whether advanced age compromises cellular reprogramming.

Here, we examined the feasibility of iPS derivation from epidermal keratinocytes from elderly T2D patients, analyzed molecular and cellular events associated with nuclear reprogramming, and determined differentiation propensities of derived pluripotent cells. Keratinocytes from T2D patients offered a reproducible source for patient-specific iPS generation, proficient in yielding insulin-producing islet-like progeny, through suppressed senescence-related pathways.

## RESULTS

### Reprogramming of human keratinocytes

**Figure 1:**
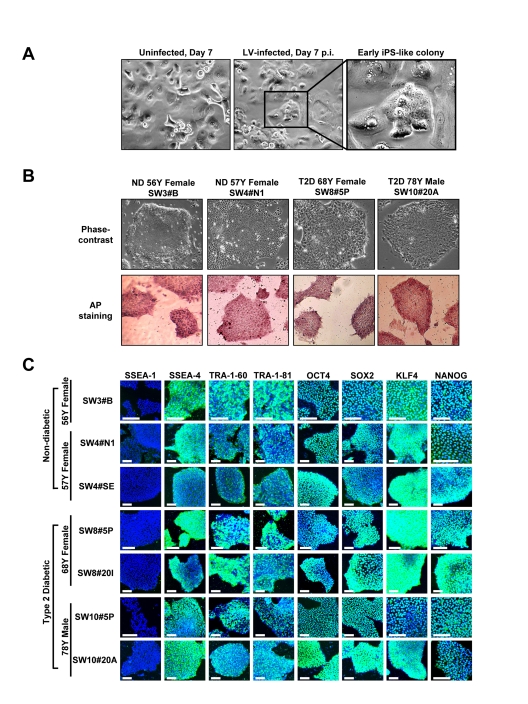
Expression of pluripotency-associated markers in HK-derived iPS clones (**A**) Early-passage HK cells (left panel) were infected with lentivirus (LV) vector encoding OCT4, SOX2, KLF4 and c-MYC. Seven days post-infection (center panel), early iPS-like colonies were detected (right panel in higher magnification). (**B**) HK-derived iPS clones were either derived from patients who were non-diabetic (ND) or type 2 diabetic (T2D). iPS clones, cultured under feeder-free conditions, exhibited human ES-like morphologies, while expressing high levels of alkaline phosphatase (AP). (**C**) Patient HK-derived iPS clones were further characterized through immunocytochemistry analysis using a panel of pluripotency markers. All clones were negative for SSEA-1 expression, while staining positive for pluripotency markers SSEA-4, TRA-1-60, TRA-1-81, OCT4, SOX2, KLF4 and NANOG. Scale bars represent 100 μm.

Lentiviral vectors encoding human OCT4, SOX2, KLF4 and c-MYC, at an approximate multiplicity of infection of 5 each, transduced early passage human keratinocytes (HK cells) derived from 56 to 78 year-old individuals with or without T2D. Under serum-free and feeder-free conditions, within 1 to 2 weeks after viral vector infection, small reprogrammed colonies, characterized by a sharp-edged, flat, tightly-packed morphology, were apparent (Figure 1A). Individual colonies were picked based on size and morphology at 3 to 5 weeks after viral transduction, and expanded. Structurally derived clones resembled human ES or fibroblast-derived iPS cells, and expressed high levels of the stemness marker alkaline phosphatase (Figure 1B). Immunocytochemistry further validated robust expression of diverse pluripotency markers, including SSEA-4, TRA-1-60, TRA-1-81, OCT4, SOX2, KLF4 and NANOG in HK-derived iPS clones regardless of patient age and status of diabetes (Figure 1C). The obtained yield was 2 to 10 expandable clones per 10^5^ transduced cells with maintained pluripotent markers and absence of replicative crisis even at 7 months post-initial vector infection (up to passage 60).

### Differentiation propensity of derived iPS cells

**Figure 2:**
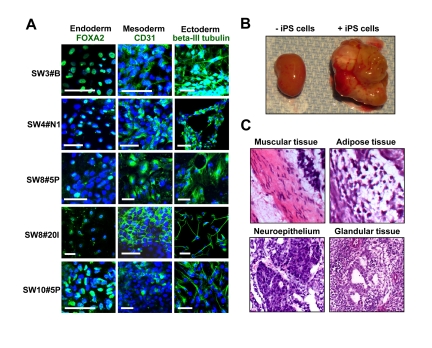
Pluripotency of HK-derived iPS cells verified through spontaneous differentiation *in vitro* and *in vivo* (**A**) HK-derived iPS clones were analyzed via immunocytochemistry for lineage markers for three germ layers (endoderm, mesoderm and ectoderm). Scale bars indicate 50 μm. (**B**) Transplant of HK-derived iPS cells into the kidney capsule of SCID-beige mice resulted in teratoma formation. Pictures of harvested kidneys (with or without iPS transplant) are shown. (**C**) H&E staining demonstrated multiple lineages within the complex architecture of the tumor, including muscle, adipose, immature neuroepithelium and glandular tissues.

HK-derived iPS clones from diabetic and non-diabetic patients spontaneously differentiated *in vitro* into cells of all three germ layers within embryoid body (EB) formations (Figure 2). In line with acquired pluripotency, HK-derived iPS cells differentiated into ectoderm (beta-III tubulin), endoderm (FOXA2) and mesoderm (CD31) as detected by immunostaining for lineage-specific markers (Figure 2A). Of note, clonal - rather than inter-patient - variations in differentiation propensities were observed within the tested cohort (Figure 2A). Moreover, *in vivo*, HK-derived iPS cells, transplanted under the kidney capsule of SCID-beige mice at a dose of 1 million cells, gave rise to 1-2 cm outgrowth within 4 weeks (Figure 2B). Tissue histology revealed iPS differentiation into mesoderm lineages indicated by muscle and adipocytes (Figure 2C), ectoderm lineages denoted by neuroepithelium-like tissues (Figure 2C), and endoderm lineages composed of glandular tissue (Figure 2C). These data document multilineage propensity of HK-derived iPS cells from both diabetic and non-diabetic patients across tested age groups.

### Genome-wide transcriptome switch underlies transition to induced pluripotency

**Figure 3:**
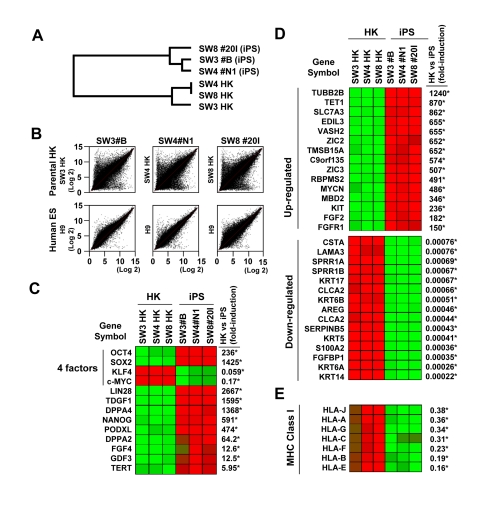
Variations in gene expression profile upon induced pluripotency (**A**) Dendrogram describing the unsupervised hierarchal clustering of patient-derived HK cells and HK-derived iPS cells. (**B**) Global gene expression patterns of HK-derived iPS clones were compared with their parental HK cells (upper panels), or with that of human embryonic stem cells (H9, lower panels, GSM190779), upon RNA microarray analysis. (**C**) Heatmap showing the up-regulation (red) and down-regulation (green) of pluripotency-associated genes in HK- and HK-derived iPS clones. The four factors used to induce pluripotency are indicated. The changes in gene expression levels in iPS cells, relative to those in parental HK cells, were calculated using microarray data from three parental HK cells and three HK-derived iPS cells, and shown as fold-induction in iPS cells. Statistically significant changes are indicated by asterisks (p<0.05). Notably, HK cells originally expressed high levels of endogenous KLF4 and c-MYC, resulting in down-regulation of these two key reprogramming factors in derived iPS cells. (**D**) Heatmap showing the top 15 genes which were up-regulated (upper panel) or down-regulated (lower panel) upon reprogramming. Statistically significant changes are indicated by asterisks (p<0.05). (E) Comparison of the major histocompatibility complex (MHC) class I gene expression profiles between HK and iPS cells. Statistically significant changes are indicated by asterisks (p<0.05).

Unbiased scan of the genome-wide transcriptome revealed distinct global gene-expression patterns in parental HK versus HK-derived iPS clones (Figure 3). The dendrogram of unsupervised one-way hierarchical clustering analysis demonstrated that HK-derived iPS cells from different patients clustered together, and branched out from its parental origin (Figure 3A). Consistent with the acquisition of a pluripotent transcriptome, gene expression patterns of HK-derived iPS cells were overall similar to those of human ES H9 cells, and different from parental counterparts (Figure 3B). Induction of key pluripotency genes, such as OCT4, SOX2, NANOG, LIN28, telomerase (TERT), DPPA4 and PODXL, were also evident in iPS clones (Figure 3C). Further analysis revealed upon reprogramming significantly up-regulated proto-oncogenes (N-MYC and KIT), pluripotency-maintenance factor FGF-2 and the receptor for FGF-2 (FGFR1), whereas cytoskeletal and keratin-encoding genes were down-regulated across HK-derived iPS clones (Figure 3D). Similar to ES cells, which are known to express minimal levels of MHC class I genes, HK-derived iPS cells showed marked down-regulation of these genes (Figure 3E). Bioinformatic analysis of transcriptome data identified pathways involved in epithelial-to-mesenchymal transition and cytoskeletal remodeling as most significantly affected networks in response to reprogramming of HK cells, in line with genuine redirection of cell fate (data not shown). No notable difference was observed in the transcriptome of iPS clones from non-diabetic and diabetic patients.

### Ultrastructural remodeling induced by reprogramming

**Figure 4:**
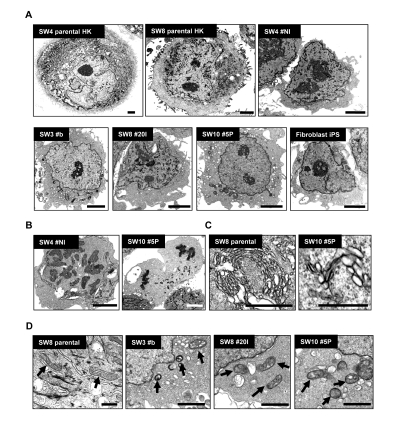
Morphological variations of patient-derived iPS cells upon reprogramming (**A**) High-resolution electron micrographs of HK cells before (SW4 parental HK and SW8 parental HK) and after (SW4 #N1, SW3 #B, SW8 #20I and SW10 #5P) induced pluripotency. Representative micrograph of a verified fibroblast-derived iPS cell is also included. Scale bars represent 2 μm. (**B**) Mitotic events of two iPS clones were shown (left panel in metaphase; right panel in anaphase). Scale bars represent 2 μm. (**C**) Endoplasmic reticulum and the Golgi structures in HK and HK-derived iPS cells are shown. Scale bars represent 0.5 μm. (**D**) Mature mitochondria with well-developed cristae in parental HK cells (SW8 parental) and immature mitochondria in iPS clones (SW3 #B, SW8 #20I and SW10 #5P) are indicated by arrows. Keratin intermediate filaments in parental HK cells are indicated by arrowheads. Scale bars represent 0.5 μm.

Electron microscopy demonstrated marked difference in the size of derived iPS compared to parental HK (Figure 4). Parental HK cells were 25 to 40 μm in diameter, while derived iPS cells were 10 to 15 μm, characterized by scant cytoplasm and regularly condensed chromatin (Figure 4A) with frequent mitotic events (Figure 4B). The cytosol of HK cells was densely packed with membrane-bound organelles (Figure 4C, left panel) and keratin intermediate filaments. In sharp contrast, widely distributed, relatively poorly developed endoplasmic reticulum and Golgi stacks were found in iPS clones (Figure 4C, right panel). In HK cells, mitochondria appeared mainly tubular-shaped and showed well-developed cristae. In contrast, mostly globular immature mitochondrial remnants, characterized by unorganized cristae, were found in HK-derived iPS cells (Figure 4D) as in verified fibroblast-derived iPS clones [16, 27] (Figure 4A). No notable difference was observed in morphologies of mitochondria between iPS clones from non-diabetic and diabetic patients.

### Reprogramming down-regulates mitochondria/oxidative stress signaling pathway

The copy number of mitochondrial DNA before and after reprogramming showed a 30 to 60% reduction in the abundance of mitochondrial DNA in iPS compared to HK cells (Supplementary Figure S1). Immuno-staining with mitochondrial probes detected mitochondria-specific signals in individual iPS cells (Supplementary Fig S1), while no significant changes in expression of nuclear-encoded mitochondrial biogenesis factors (Supplementary Figure S1). Selected genes involved in the TCA cycle, such as ACO2, SDHA and FH, were down-regulated by nuclear reprogramming (Supplementary Figure S1). Transcriptome analysis further revealed that genes encoding the mitochondrial/oxidative stress response pathway are highly expressed in HK cells from elderly patients, yet markedly down-regulated in derived iPS cells (Supplementary Figure S1). Reduced transcription following reprogramming was particularly evident in major antioxidant enzymes [28], such as catalase CAT and GPX1 (Supplementary Figure S1), suggesting reversal of senescence cellular markers.

### Reprogramming induces telomere elongation and down-regulates genes involved in senescence

**Figure 5:**
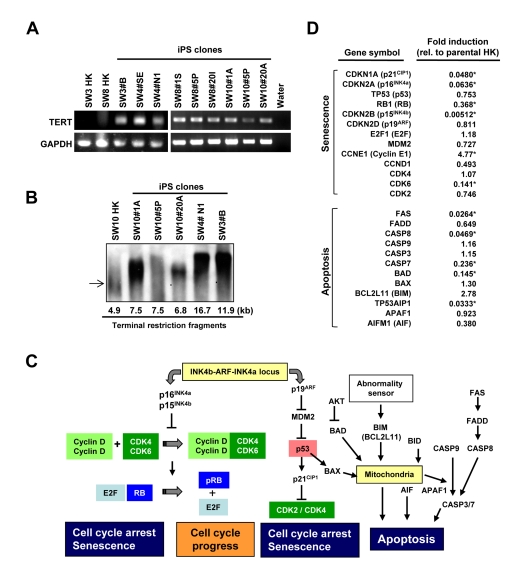
Comparison of telomerase activity, cellular senescence and programmed cell death in HK cells before and after induced pluripotency (**A**) RT-PCR analysis of TERT-specific transcripts in parental HK cells and iPS clones. GAPDH was used as control. (**B**) Telomere lengths in HK and HK-derived iPS cells were determined by the terminal restriction fragment lengths. Southern blot analysis and corresponding telomere fragment lengths derived from densitometric quantification are shown. (**C**) Schematic representation of key senescence- and apoptosis-regulating pathways. (**D**) Changes in expression levels of key genes, involved in cellular senescence or apoptosis, were determined using the microarray data of three parental HK cells and three HK-derived iPS cells, and fold induction of individual genes in iPS cells, relative to those in parental HK cells, are shown. Statistically significant changes are indicated by asterisks (p<0.05).

RT-PCR verified increased levels of TERT-specific transcripts in HK-derived iPS cells (Figure 5A). In fact, the telomere restriction fragment (TRF) assay further demonstrated that HK-derived iPS cell lines display longer telomeres than parental HK cells (Figure 5B), indicating reprogramming induced telomere elongation regardless of diabetes status. Comparison of the transcriptome between three parental HK cells (SW3-HK, SW4-HK and SW8-HK) and derived iPS clones (SW3 #B, SW4 #N1 and SW8 #20I) revealed significant down regulation (p < 0.05) of senescence/apoptosis-associated genes (Figure 5C), including p16^INK4a^ and p15^INK4b^ in the p16^INK4a^/RB pathway, and p21^CIP1^ in the p19^ARF^/p53 pathway, and proapoptotic genes, including FAS, CASP8, CASP7, BAD and TP53AIP1 (Figure 5D). Thus successful cellular reprogramming of somatic cells from elderly patients is associated with suppression of key senescence- and apoptosis-related pathways in diabetic and non-diabetic patients.

### Proficiency of HK-derived iPS cells to yield insulin-producing islet-like progeny

**Figure 6:**
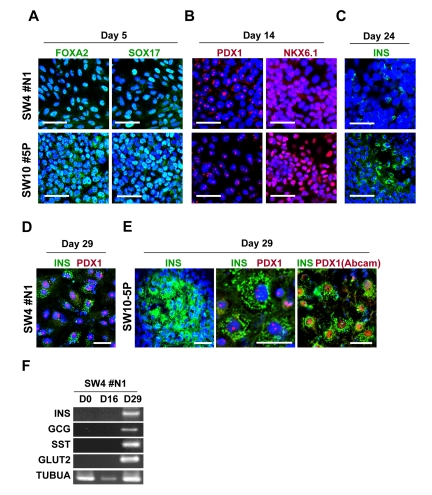
Guided *in vitro* differentiation of patient iPS cells into insulin-producing islet-like cells iPS cells, differentiated through step-wise differentiation, were analyzed by immunocytochemistry for stage-specific markers at day 5 (**A**), 14 (**B**), 24 (**C**) and 29 (**D** and **E**). Scale bars indicate 50 μm for **A**, **B**, **C** and **E** (left panel), 10 μm for **D** and E (middle and right panels) and an alternative antibody (Abcam, #ab47267) against PDX1 is shown in **E** (right panel). (**F**) RT-PCR analysis of the mRNA of SW4#N1 clone, harvested at differentiation day 0, 16 and 29, confirmed the expression of insulin (INS), glucagon (GCG), somatostatin (SST), glucose transporter 2 (GLUT2) on day 29. α-tubulin was used as control (TUBUA).

HK-derived iPS clones were initially induced to form definitive endoderm by treatment with activin A and Wnt3a for 1 day followed by culture in activin A and 2% FBS for 4 additional days. Immunostaining revealed efficient induction in iPS-derived cells of SOX17 and FOXA2, markers of definitive endoderm (Figure 6A).

Similar results were observed with iPS clones generated from diabetic or non-diabetic patients. Next, we evaluated the efficiency of definitive endoderm transformation into pancreatic endoderm. As shown in Figure 6B, prominent nucleus-localized signals for pancreatic endoderm, namely PDX1 and NKX6.1, were found in iPS-derived cells at day 14 of differentiation. No notable difference was found among iPS clones from non-diabetic and diabetic patients. These results indicate successful induction of pancreatic endoderm from HK-iPS-derived definitive endoderm. In the presence of DAPT and GLP-1, iPS-derived pancreatic endoderm cells were further differentiated for 6 days, followed by maturation in HGF, IGF-1, and GLP-1 for additional 8 days. By day 24, insulin-producing cells were sporadically detected in iPS-derived progeny (Figure 6C), while more prominent immunostaining for insulin evident after final maturation at day 29 (Figure 6D and E). Similar to pancreatic beta cells which co-express insulin and PDX1, the majority of iPS-derived insulin-expressing cells showed nuclear-localized PDX1 signals (Figure 6D and E). High levels of intracellular C-peptide (250-290 pM), a byproduct of proinsulin protein process-sing, were detected in iPS progeny by ELISA, while RT-PCR revealed positive gene expression of key pancreatic factors, including insulin (INS), glucagon (GCG) and somatostatin (SST), and glucose transporter 2 (GLUT2) (Figure 6F). Thus, HK-derived iPS cells differentiate into hormone-producing pancreatic islet-like cells.

## DISCUSSION

The present study reports derivation of iPS cells from T2D patients. Human keratinocytes provided the starting somatic tissue reprogrammed here, under serum/feeder-free conditions, into genuine pluripotent derivatives proficient in generating insulin-producing islet-like progeny. Dedifferentiation of epidermal keratinocytes from elderly patients was driven by induction of stemness transcription factors, and associated with suppression of senescence/apoptosis gene sets. Lineage specification mimicked natural pancreatic development encompassing staged differentiation from definitive endoderm to hormone-producing islet-resembling phenotype. Thus, derivation of proficient iPS cells from elderly T2D patients is feasible, expanding the spectrum of disease entities amenable to somatic cell fate redirection.

To date, disease-specific iPS cells have been established from a series of diverse human disease conditions, including amyotrophic lateral sclerosis, type 1 diabetes, Huntington's and Parkinson's disease, muscular dystrophy, Fanconi anemia, Down syndrome, dyskeratosis congenita and gyrate atrophy [14, 26, 29-34]. It is notable that iPS cells from patients with spinal muscular atrophy, familial dysautonomia and LEOPARD syndrome recapitulated the respective disease phenotypes upon redifferentiation [35-37], underscoring the value of derivation of patient-specific iPS cells for disease modeling and molecular diagnostics. Although T2D is one of the most prevalent degenerative diseases, derivation of T2D-specific iPS cells has not yet been documented. Moreover, the impact of aging on nuclear reprogramming remains elusive. Here, we demonstrate feasibility and reproducibility of iPS derivation from elderly T2D patients, extending the disease-specific collection of validated human iPS clones. The uniqueness of T2D among human degenerative diseases is the high prevalence within an exponentially growing elderly population and association with complications across distinct tissue types including severe retinopathy, nephropathy, neuropathy and cardiovascular diseases. T2D-specific iPS cells, derived herein, would thus provide a previously unavailable platform to study the mechanisms of T2D progression and T2D-associated complications.

Surgical skin specimens provided herein epidermal keratinocytes used as the somatic source for efficient and rapid iPS derivation. Epidermal keratinocytes have been used for skin transplant, and more recently proposed as a source for bioengineering pluripotent stem cells following the successful reprogramming from normal human foreskin or plucked hair from children or young adults [25]. In addition to the inherently high expression of selective stemness genes c-MYC and KLF4 which may facilitate cell fate redirection, keratinocytes were amenable here to culture in xeno-free systems, and responsive to feeder-free reprogramming, rendering HK cells a robust source for iPS derivation from elderly T2D patients.

Studies have established that cellular senescence impairs nuclear reprogramming in murine primary cultures [38-40] or human cell lines [39, 41]. Accordingly, somatic cells from old mice are resistant to reprogramming compared to young mice [39]. Conversely, siRNA-mediated inhibition of p16^I^^NK^^4a^, p19^Arf^ or p53 improves reprogramming efficiency [38-40, 42]. Moreover, genetic ablation of p16^INK4a^ or p53 or immortalization with TERT overexpression significantly improves reprogramming efficiency [38, 40]. Present data revealed that reprogramming of HK cells from elderly patients is accompanied with telomere elongation and suppression of senescence- and apoptosis-related genes, suggesting induction of a rejuvenated state, in line with successful derivation of IPS cells from elderly individuals [30]. Marked suppression of p16^INK4a^, but not p19^ARF^, in HK-derived iPS cells supports the notion that p16^INK4a^, rather than p19^ARF^, is a main regulator of reprogramming in human cells [39]. Since a 20-fold down-regulation of p21^CIP1^ was found in HK-derived iPS cells, it could be speculated that the p19^ARF^/p53 senescence pathway is suppressed at the p21^CIP1^ stage, rather than at p19^ARF^. Although we were successful in generating iPS cells from elderly patients, it is plausible that RNAi-mediated down-regulation of p16^I^^NK^^4a^, p21^CIP1^, and/or CASP8 in HK cells could further improve iPS derivation efficiency. In addition, we also found broad suppression of genes involved in the senescence-related, mitochondrial/oxidative stress pathway in HK-derived iPS cells. Overall, the present results imply an induced state of rejuvenation in iPS cells derived from elderly patients, with the Ink4a/Arf/p53 signaling critical for somatic cell reprogramming.

A promising strategy to overcome shortage of transplantable islet cells is to generate insulin-secreting cells from patient-derived iPS cells [43). Here, through guided differentiation with ILV and GLP-1, we differentiated HK-derived iPS cells into insulin-producing cells *in vitro*. This is the first report demonstrating successful generation of insulin-producing cells from T2D-specific iPS cells. Of note, p16^INK4a^ induces an age-dependent decline in islet regenerative potential [44]. Transgenic mice with overexpressed p16^INK4a^ show decreased islet proliferation, while increased islet regeneration is observed in p16^INK4a^-deficient old mice [44]. Given the suppression of senescence-related genes, including p16^INK4a^, in HK-derived iPS cells observed here, the use of iPS cells for islet regeneration appears to be a rational strategy, especially for autologous cell therapy for elderly patients.

In summary, we demonstrate the feasibility and reproducibility of iPS cell derivation from elderly patients with T2D. Reprogramming of HK cells was accompanied by morphological changes, induction of endogenous pluripotency genes, telomere elongation, and down-regulation of senescence- and apoptosis-related genes. Notably, stepwise differentiation with ILV and GLP-1 achieved successfully differentiation of T2D-specific iPS cells into insulin-producing islet-like cells. Thus, reprogramming of keratinocytes from elderly T2D patients yields proficient iPS cells through induction of a senescence privileged status. T2D-specific iPS cells would provide a versatile platform for disease modeling and regenerative applications.

## METHODS

Protocols approved by Mayo Clinic Institutional Review Board and Institutional Animal Care and Use Committee.

### Human keratinocytes

Skin specimens from surgical pathology from non-diabetic and type 2 diabetic (T2D) individuals were enzymatically processed. Using sterile techniques, skin samples were incubated overnight at 4°C in dispase (25 U/ml) to cleave epidermis from dermis. The epidermal layer was then placed into a recombinant trypsin/EDTA solution (Invitrogen, Carlsbad, CA,) and incubated for 30 min at 37°C. Trypsin/EDTA was neutralized with a trypsin inhibitor (Invitrogen, Carlsbad, CA) and epidermal pieces pipetted to release epidermal cells. The suspension was then passed through a 70 μm cell strainer and pelleted. Cell viability was determined by the trypan blue exclusion method. Cells were seeded in a plate coated with an animal component-free (ACF) coating matrix (Invitrogen). Selective trypsinization removed fibroblasts at ~6 min, while human keratinocytes (HK) were dissociated at ~20 min. HK cell populations were then grown in EpiLife Medium and S7 growth supplement (Invitrogen, Carlsbad, CA) in 5% CO_2_ and 95% air at 37°C. HK cells were maintained semi-confluent in low calcium media.

### Reprogramming

Lentiviral vectors, pSIN-OCT4, pSIN-SOX2, pSIN-KLF4 and pSIN-cMYC were manufactured as previously described to express pluripotency factors from an internal spleen focus-forming virus (SFFV) promoter [45]. HIV vectors were produced by transient transfection of 293T cells. To minimize calcium-mediated differentiation of HK cells during vector infection, lentiviral vectors were concentrated by ultracentrifugation and re-suspended in PBS [46]. Lentiviral titers were determined by immunostaining [45]. Human HK cells were grown *in vitro* in ACF EpiLife Medium in a matrix-coated plate. Cultures were transduced overnight with human OCT4, SOX2, KLF4 and cMYC expressing lentiviral vectors [45). Culture supernatants were replaced daily with ACF media. At 4 days after vector infection, media was changed to HEScGRO medium (100 ml, Millipore, Billerica, MA) supplemented with mTeSR-1 maintenance media (25 ml, Stemcell Technologies, Vancouver, BC, Canada) [16]. One to two weeks after vector infection, reprogrammed cells began to form colonies displaying stem cell morphology [16]. At three to four weeks after vector infection, cultures were treated with Cell Dissociation Buffer (Invitrogen, Carlsbad, CA) for 5 to 10 min to help lift clones picked by a P200 pipette, and placed in BD Matrigel (BD Biosciences, San Jose, California) coated 96-well plates. To prevent spontaneous differentiation, the iPS culture medium was replaced daily and differentiated cells in cultures manually removed. As clones grew, cultures were expanded into larger culture plates for further characterization. iPS clones were preserved using Xeno-FREEze™ Human Embryonic Stem Cell Freezing Medium (Millipore, Billerica, MA). For spontaneous differentiation, iPS clones were dissociated using collagenase IV (Stemcell Technologies) for 30 min and plated on low adhesion plates in basal HEScGRO medium without bFGF. Embryoid bodies (EBs) were cultured as suspensions for 7-14 days, and grown adherent in DMEM with 20% FBS for additional 7-14 days.

### Differentiation of iPS cells into insulin-producing cells

iPS clones were treated with 25 ng/ml Wnt3a (R&D systems) and 100 ng/ml activin A (Peprotech) in advanced RPMI (Invitrogen) with Pen/Strep for 1 day, followed by treatment with 100 ng/ml activin A in advanced RPMI supplemented with 0.2% fetal calf serum (FCS) (Invitrogen) for two days. Next, cells were cultured in high glucose DMEM (Invitrogen), supplemented with 20% (v/v) advanced RPMI medium containing 50 ng/ml FGF10 (R&D systems), 0.25 μM KAAD-cyclopamine (CYC), and 2% FCS for 2 days. Cells were then treated with 50 ng/ml FGF10, 0.25 μM CYC, and 2 μM all-*trans* Retinoic Acid (RA) (Sigma) in high glucose DMEM (Invitrogen) supplemented with 20% advanced RPMI, Pen/Strep, 1x B27 supplement (Invitrogen) for 4 days. Cells were then cultured in 50 ng/ml FGF10, 300 nM ILV (Axxora) and 55 nM GLP-1 (Sigma) in DMEM (high glucose) supplemented with 20% advanced RPMI and 1x B27 for 4 days. Differentiation medium including 10 μM DAPT (Sigma) and 55 nM GLP-1 in DMEM (high glucose) with 20% advanced-RPMI and 1x B27 was used to culture cells for the next 6 days. Finally, cells were cultured in 50 ng/mL hepatocyte growth factor (HGF) (R&D systems), 50 ng/ml insulin-like growth factor 1 (IGF-1) (R&D systems) and 55 nM GLP-1 in CMRL-1066 medium (Invitrogen) with 1 × B27 for 8 days.

### Immunostaining

For immunostaining, iPS cells were fixed for 20 min at room temperature (RT) in 4% paraformaldehyde (PFA), washed in PBS and blocked for 30 min in 5% FBS PBST (PBS with 0.1% Tween-20 (Sigma) and 5% FBS). Cells were stained with primary antibodies overnight at 4°C, rinsed by PBS, and incubated with secondary antibodies for 1 h at room temperature. Separately, cells at different stages of differentiation were fixed and stained with primary and secondary antibodies. Primary and secondary antibodies used for characterization were: SSEA-1, SSEA-4, TRA-1–60 TRA-1-81 (Millipore #SCR001), OCT4 (Cell Signaling Technology #2750), SOX2 (Cell Signaling Technology #2748), KLF4 (Abcam #ab26648) and NANOG (Abcam #ab21624), anti-SOX17 (R&D Systems #MAB1924), anti-HNF3 beta/FOXA2 (Millipore #07-633), anti-PDX1 (Santa Cruz Biotechnology#sc-25403 and Abcam #ab47267), and anti-insulin (Sigma # I2018). Texas Red-conjugated anti-rabbit IgG (Jackson Laboratories # 711-075-152), Texas Red-conjugated anti-mouse IgG (Jackson Laboratories # 715-075-151), FITC-conjugated anti-rabbit IgG (Jackson Laboratories # 711-095-152), and FITC-conjugated anti-mouse IgG (Jackson Laboratories # 715-095-151) were used as secondary antibodies. DAPI was used to counter-stain nuclei. Stained cells were analyzed using confocal laser-scanning microscopy (Zeiss, LSM 510 confocal scanning laser system). Alkaline phosphatase staining was performed with an Alkaline Phosphatase Detection Kit (Millipore). Antibodies FOXA2 for endoderm, beta III tubulin (Abcam # ab41489) for ectoderm and CD31 (Santa Cruz Biotechnology # sc-1506) for mesoderm were used to immunostain embryoid body-derived cells.

### *In vivo* differentiation of iPS cells

SCID-beige mice were anesthetized, and the kidney exposed for iPS transplantation under the kidney capsule. To this end, a small incision was made in the kidney capsule and a blunt needle was used to create a pocket under the kidney capsule. Following iPS cell injection, the kidney was placed back into the abdomen, and the incision closed. Mice were maintained for 4 weeks and sacrificed for harvesting normal and iPS-transplanted kidneys. OTC-embedded frozen tissues were cryo-sectioned for H&E staining.

### Gene expression

For amplification of mitochondrial DNA, mitochondria-specific primer pairs (CYTB, CCTAGCCATGCACTACTCACCAGACGCCT, CTG TCTACTGAGTAGCCTCCTCAGATTC; and NADH, TCACCAAAGAGCCCCTAAAACCCGCCACATCTA, TAAGGGTGGAGAGGTTAAAGGAGC) were used. For RT-PCR analysis, total RNA was isolated using TRIzol (Invitrogen) and reverse transcription was performed with oligo (dT) primer using RNA to cDNA EcoDry (Clontech). Platinum Taq DNA polymerase (Invitrogen) and primer pairs for TERT (TGTGCACCAACATCTACAAG, GCGTTCTTGGCT TTCAGGAT), INS (AGCCTTTGTGAACCAACACC, GCTGGTAGAGGGAGCAGATG), SST (GTACTTCT TGGCAGAGCTGCTG, CAGAAGAAATTCTTGCAG CCAG), GCG (AGGCAGACCCACTCAGTGA, AACAATGGCGACCTCTTCTG), GLUT2 (GCTACC GACAGCCTATTCTA, CAAGTCCCACTGACATGA AG) and α-tubulin (AAGAAGTCCAAGCTGGAGTTC, GTTGGTCTGGAATTCTGTCAG) were used for the reaction. Separately, total RNA was isolated using TRIzol (Invitrogen) and further purified using RNeasy Plus spin columns (QIAGEN). Turbo DNA-free DNase (Ambion, Austin, TX) was used to digest all genomic DNA that could lead to false positive gene expression results. RNA quantity and purity were measured with a Nanodrop spectrophotometer (Thermo Scientific, Wilmington, DE) and RNA integrity was determined using the Agilent 2100 Bioanalyzer (Santa Clara, CA). Microarray analysis was performed using the Affymetrix HG-U133 Plus2 GeneChip Array platform (Affymetrix, Santa Clara, CA). Data were preprocessed using MicroArray Pre-Processing workflow and hierarchical clustering was performed by Pearson Dissimilarity. For comparison of transcriptome data between pre- and post-reprogramming, the data set of parental HK cells from three patients (SW3, SW4 and SW8) were compared with those of three iPS clones from the same patients (SW3 #B, SW4 #N1 and SW8 #20I). Student's t-test was performed to assess significance (p<0.05) in normalized gene expression levels between HK and HK-derived iPS clones. The Heatmap Builder software (kindly provided by Dr. Euan Ashley, Stanford University) was used to generate the heatmap for the transcriptome data set. Enrichment analysis was also performed to match gene IDs in functional ontologies. The registered GEO transcriptome information (GSM551202, human ES H9 cell transcriptome) was used as reference.

### Telomere assay

Total genomic DNA was isolated from patient-derived HK and iPS cells using QIAGEN DNeasy Blood & Tissue Kit. Telomere length was determined using TeloTAGGG telomere length assay (Roche). Genomic DNA digestion, Southern blotting and chemiluminescence detection was performed as per established protocols. Densitometric analysis was performed on Adobe Photoshop and terminal restriction fragment lengths determined by Σ(OD_i_)/Σ(OD_i_/L), where OD_i_ and L were the optical density and length of fragment, respectively.

## SUPPLEMENTAL FIGURES

Supplementary Figure S1 Mitochondrial and oxidative-stress response gene expression in induced pluripotency (**A**) Relative cytochrome B (CYTB) and NADH mitochondrial DNA (mtDNA) copy numbers before (parental) and after (iPS) reprogramming. mtDNA copy numbers were normalized to total genomic DNA and represented as a percentage of parental cell mtDNA copy number. (**B**) Immunocytochemistry analysis of iPS clone SW4 #N1 with mitochondrial marker AIF and (**C**) iPS clones SW4 #N1 and SW10 #5P with MitoTracker (Molecular Probes) staining. (**D**) Heatmap demonstrating up (red) and down-regulation (green) of genes involved in mitochondrial biogenesis upon reprogramming. No statistically significant change was observed in any of the genes listed. (**E**) Heatmap of expression profiles for genes involved in glycolysis, anaerobic glycolysis and citric acid cycle were compared between parental HK and HK-derived iPS cells. Statistically significant changes are indicated by asterisks (p<0.05). (**F**) RNA expression profiles of genes involved in the mitochondrial/oxidative stress response pathway between parental HK and iPS cells are shown. Statistically significant changes are indicated by asterisks (p<0.05).
