# Supplementary figures and images for: Reprogrammed keratinocytes from elderly type 2 diabetes patients suppress senescence genes to acquire induced pluripotency
Source: Aging (Albany NY). 2012 Feb 4;4(1):60–73. doi: 10.18632/aging.100428 (PMC3292906; doi:10.18632/aging.100428)

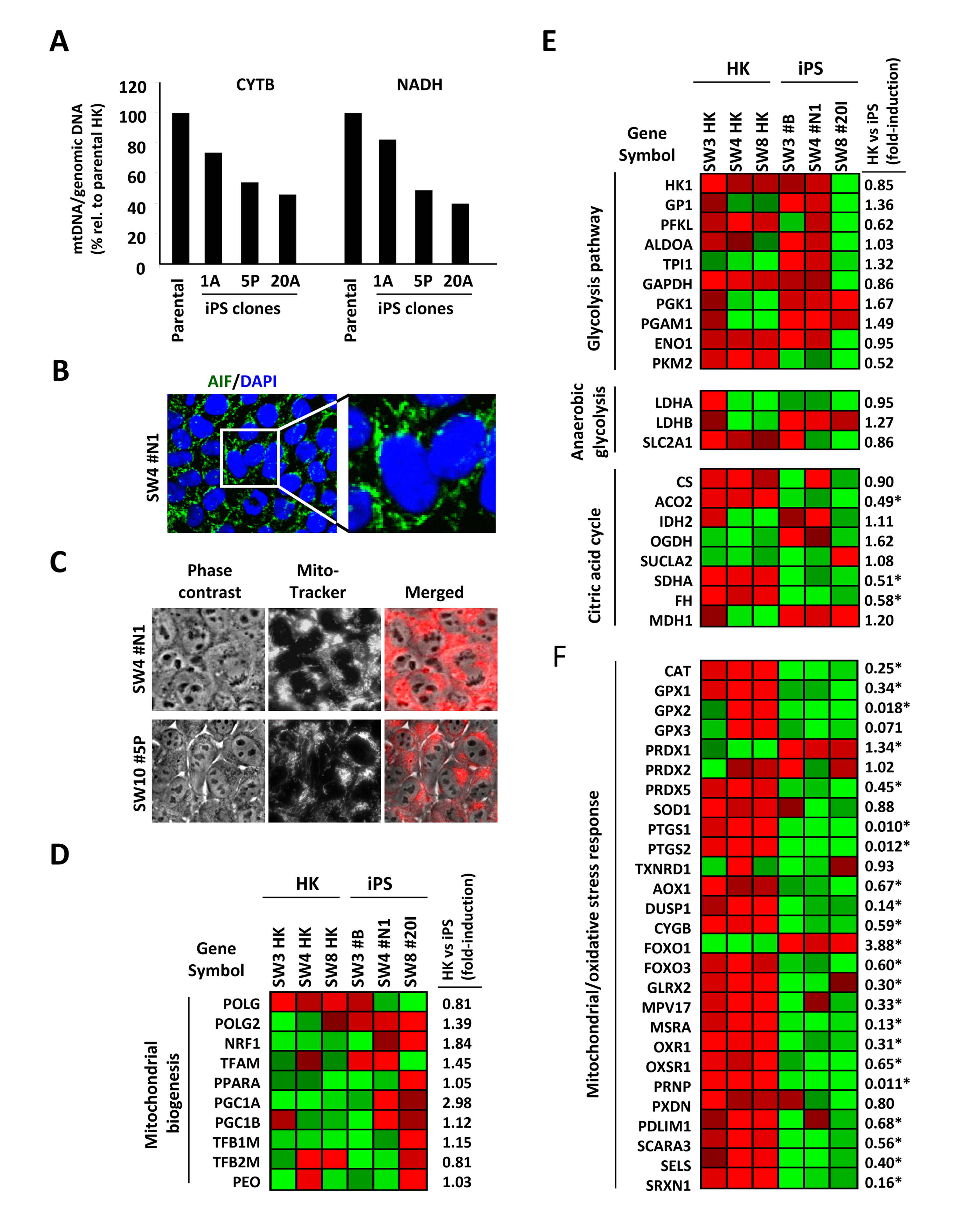

Supplement: Supplementary Figure S1 — (A) Relative cytochrome B (CYTB) and NADH mitochondrial DNA (mtDNA) copy numbers before (parental) and after (iPS) reprogramming. mtDNA copy numbers were normalized to total genomic DNA and represented as a percentage of parental cell mtDNA copy number. (B) Immunocytochemistry analysis of iPS clone SW4 #N1 with mitochondrial marker AIF and (C) iPS clones SW4 #N1 and SW10 #5P with MitoTracker (Molecular Probes) staining. (D) Heatmap demonstrating up (red) and down-regulation (green) of genes involved in mitochondrial biogenesis upon reprogramming. No statistically significant change was observed in any of the genes listed. (E) Heatmap of expression profiles for genes involved in glycolysis, anaerobic glycolysis and citric acid cycle were compared between parental HK and HK-derived iPS cells. Statistically significant changes are indicated by asterisks (p<0.05). (F) RNA expression profiles of genes involved in the mitochondrial/oxidative stress response pathway between parental HK and iPS cells are shown. Statistically significant changes are indicated by asterisks (p<0.05). [file aging-04-060-s001.tif]
